# The Role of Nitrogen Fixation in Cyanobacterial Bloom Toxicity in a Temperate, Eutrophic Lake

**DOI:** 10.1371/journal.pone.0056103

**Published:** 2013-02-06

**Authors:** Lucas J. Beversdorf, Todd R. Miller, Katherine D. McMahon

**Affiliations:** 1 Department of Civil and Environmental Engineering, University of Wisconsin-Madison, Madison, Wisconsin, United States of America; 2 Department of Bacteriology, University of Wisconsin-Madison, Madison, Wisconsin, United States of America; 3 Zilber School of Public Health, University of Wisconsin-Milwaukee, Milwaukee, Wisconsin, United States of America; Argonne National Laboratory, United States of America

## Abstract

Toxic cyanobacterial blooms threaten freshwaters worldwide but have proven difficult to predict because the mechanisms of bloom formation and toxin production are unknown, especially on weekly time scales. Water quality management continues to focus on aggregated metrics, such as chlorophyll and total nutrients, which may not be sufficient to explain complex community changes and functions such as toxin production. For example, nitrogen (N) speciation and cycling play an important role, on daily time scales, in shaping cyanobacterial communities because declining N has been shown to select for N fixers. In addition, subsequent N pulses from N_2_ fixation may stimulate and sustain toxic cyanobacterial growth. Herein, we describe how rapid early summer declines in N followed by bursts of N fixation have shaped cyanobacterial communities in a eutrophic lake (Lake Mendota, Wisconsin, USA), possibly driving toxic *Microcystis* blooms throughout the growing season. On weekly time scales in 2010 and 2011, we monitored the cyanobacterial community in a eutrophic lake using the phycocyanin intergenic spacer (PC-IGS) region to determine population dynamics. In parallel, we measured microcystin concentrations, N_2_ fixation rates, and potential environmental drivers that contribute to structuring the community. In both years, cyanobacterial community change was strongly correlated with dissolved inorganic nitrogen (DIN) concentrations, and *Aphanizomenon* and *Microcystis* alternated dominance throughout the pre-toxic, toxic, and post-toxic phases of the lake. Microcystin concentrations increased a few days after the first significant N_2_ fixation rates were observed. Then, following large early summer N_2_ fixation events, *Microcystis* increased and became most abundant. Maximum microcystin concentrations coincided with *Microcystis* dominance. In both years, DIN concentrations dropped again in late summer, and N_2_ fixation rates and *Aphanizomenon* abundance increased before the lake mixed in the fall. Estimated N inputs from N_2_ fixation were large enough to supplement, or even support, the toxic *Microcystis* blooms.

## Introduction

Anthropogenic eutrophication of freshwaters has led to altered ecosystem function and structure, water quality degradation, and economic loss [1]. Destruction of ecosystem goods and services as a result of eutrophication has resulted in over $1 billion in expenditures annually within the United States alone [2]. Long-term monitoring of ecosystem status (e.g. freshwater quality) has generally followed a structural approach, monitoring metrics such as total nitrogen (TN) and total phosphorus (TP) concentrations and biomass indicators such as chlorophyll-*a* [3]. More recently, a debate in the literature has emerged about the importance of N loadings relative to P loadings on inter-annual time scales [4], [5]. While N and P loading have undoubtedly contributed to an increased occurrence of harmful cyanobacterial blooms (cHABs) in freshwaters, estuaries, and coastal oceans [6], [7], very little is known about how varying nutrient fluxes will affect subgenus cyanobacterial population dynamics, and more specifically, ecosystem functions such as cyanotoxin production [8]. The cyclical nature of nutrient draw-down and recycling seems to act as a feedback loop to population dynamics, making it impossible to separate whether blooms are simply a cause or a consequence of dissolved nutrient scarcity. This is particularly true on intra-annual time scales in which toxic blooms may occur during periods when dissolved nutrients appear to be exhausted, regardless of total N and P loadings.

Cyanotoxin production has been extensively studied in laboratory-based batch and chemostat experiments. Increased toxin production has been observed under varying P [9], [10], N [11], [12], N and P [13], iron (Fe) [14], [15], and sulfur (S) concentrations [16], as well as light availability [17], and varying growth states [18], [19]. Most studies indicate that the highest toxin production occurs under the most advantageous growth conditions [20], usually after cultures have been acclimated to the desired condition. However, Downing *et al* [21] showed that growth rate was not the only factor controlling microcystin production and found that nitrate uptake was highly correlated with toxin production, whereas P uptake and carbon (C) fixation were negatively correlated. In addition, Ginn *et al* [12] revealed that the transcription factor NtcA, a global nitrogen regulator, binds to the microcystin promoter region of the *mcyA/D* genes in *Microcystis aeruginosa* and *mcyB* and *ntcA* are up regulated under N limited and starved conditions. Both of these studies are consistent with a recent proteomic study of 6 *Microcystis aeruginosa* strains (3 toxic, 3 nontoxic) in which there was a relative up regulation of the NrtA protein and down regulation of P_II_ proteins in the toxic strains [22]. Alexova *et al* [22] suggested that C fixation, N metabolism, and photosynthesis are all linked in toxic *Microcystis aeruginosa* with NtcA as the potential global regulator, but also acknowledged the limitations of using a single species, under nutrient replete conditions, as a model system.

In contrast to laboratory studies, ecosystem-based studies have attempted to correlate multiple environmental factors to the presence of cyanotoxins and/or toxic cyanobacteria in aquatic systems to elicit an ecological role for, and eventually predict, the production of these secondary metabolites. Oh *et al* [23] found that microcystin concentrations were highly correlated to phytoplankton biomass and chlorophyll-*a*. Interestingly, however, microcystin did not correlate to *Microcystis*, *Anabaena*, or cyanobacteria biomass. Wicks and Thiel [24] correlated peptide toxins with primary production coupled to several environmental factors including solar radiation, temperature, and dissolved oxygen concentrations. Kotak *et al* [25] found no relationship between microcystin-LR (MCLR) and temperature, and although insignificant, there was an inverse relationship between MCLR and nitrate concentrations. In addition, Kotak *et al* [26] found a significant negative correlation between MCLR and secchi depth and significant positive correlations between MCLR and TP, pH, and chlorophyll-*a*. Similarly in Lake Erie, Rinta-Kanto *et al* [27] found a positive correlation between TP and *mcyD*, microcystin, and *Microcystis* 16S rDNA, whereas TN, nitrate, and TN∶TP ratios were negatively correlated with *mcyD*, microcystin, and *Microcystis*. The results of studies conducted on natural communities can often be difficult to interpret due to the simultaneous presence of toxic and nontoxic species of the same genera, as well as different genera that concurrently produce the same toxin [28]. In addition, the presence of different cyanobacterial genera, as well as other eubacterial phyla, can be highly variable across nutrient gradients [8], [29], both spatially and temporally. Therefore, there is an immediate need to identify both structural- and functional-based environmental drivers of toxic species and toxin production at the appropriate time and space scales, and to better integrate the results of laboratory-based studies with field-scale observations.

Our objectives in this study were to 1) describe the timing of N_2_ fixation relative to rapid declines in dissolved inorganic nitrogen (DIN) concentration upon lake stratification, and 2) to determine if the level of N input due to N_2_ fixation could support microcystin production. To do this, we tracked N_2_ fixation rates through time and related it to DIN drawdown rates and N∶P ratios. N_2_ fixation is energetically expensive and heavily regulated. It has been shown to occur only when bacteria are starved for other sources of combined N (e.g. ammonium and nitrate), specifically when cellular concentrations of 2-oxoglutarate (2-OG) surpasses a critical threshold [30]. In addition, N_2_ fixation is important in aquatic ecosystems because it often leads to the production of “new” N. This may be in contrast to N-limited, shallow polymictic lakes where N inputs can come from regenerated benthic N and heavy precipitation events [31]. The concept of new N was developed for marine systems and refers to the subsidy of N to surface waters via upwelled nitrate or conversion of atmospheric N_2_ [32]. New N can enter the system as ammonium ions (NH_4_^+^) and dissolved organic nitrogen (e.g. glutamate and glutamine) that leak out of cells during the fixation process [33], [34], or after cell lysis and remineralization. This input of new N can often support the growth of contemporary phytoplankton communities and has been suggested in a variety of settings to stimulate subsequent blooms of benthic algae, non-N_2_ fixing cyanobacteria, and the toxic dinoflagellate, *Karenia brevis* [35], [36], [37]. In model simulations, Agawin *et al* [35] calculated that *Synechococcus* abundance was four times greater in competition experiments with the N_2_-fixing *Cyanothece* than it would have been in monoculture.

Although the importance of new N has been recognized in both oceanic and laboratory setting, to our knowledge, no studies have linked N_2_ fixation and new N production to toxic cyanobacteria in freshwater ecosystems despite the ubiquitous threat cyanotoxins have on water quality worldwide. Therefore, we estimated the amount of new N that could be supplied to the surface waters and could potentially support *Microcystis* growth and the production of toxins. We sampled Lake Mendota, Wisconsin, USA —a large, temperate dimictic lake—weekly from spring to fall over two years to assess the importance of N stress on cyanobacterial population dynamics and its potential role in toxin production. We employed a structural and functional approach by combining community analyses, toxin measurements, N_2_ fixation rates, and multivariate statistics to assess the importance of several environmental drivers both spatially and temporally.

## Materials and Methods

### Ethics statement

No specific permits were required for the described field study, nor were any specific permissions needed to sample the following locations. Lake Mendota is not privately owned, and our sampling did not involve endangered or protected species.

### Lake characteristics

Lake Mendota is a eutrophic lake located within the Six Mile and Pheasant Branch Creek watershed of south central Wisconsin (43.0995, −89.4045). It is characterized by large nutrient inputs (mostly N and P) from both agricultural and urban run-off [6], and is dimictic, mixing on average in mid-April and mid-September while strongly stratifying during the summer. It has a surface area of 39.98 km^2^ with a maximum depth of 25.3 m and a mean depth of 12.8 m. We sampled three locations in 2010 based on their spatial heterogeneity, depth, and chemical and physical differences: 1) Deep Hole (DH, 25 m), 2) Green Acres (GA, 18 m), and 3) University Bay (UB, 5 m) (Map, Figure S1.). The DH location is the site of the North Temperate Lakes-Long Term Ecological Research (NTL-LTER; http://lter.limnology.wisc.edu/) program, which measures physical, chemical, and biological parameters biweekly throughout the year, and is also the site of a moored buoy, which collects high-resolution meteorological, temperature, dissolved oxygen, and pigment data during the ice-off season. Our spatial survey took place between May 20^th^ and August 31^st^, 2010, which encompassed the time between spring and fall mixing (*n* = 42 samples total). In 2011, we sampled only the Deep Hole location in order to increase sampling frequency. Our temporal survey also covered a longer period of time extending from May 6^th^ to October 7^th^, 2011.

### Field sample collection and processing

At each location, temperature, dissolved oxygen (DO), and pH were collected at 1 m increments from the surface to the maximum depth (YSI 556MPS). Photic zone depth was defined at 1% of photosynthetically active radiation (PAR) as measured using a PAR sensor (LiCor 192SA). Integrated photic zone samples were then collected using a weighted 2-inch diameter polypropylene tube. Samples for DNA, nutrients, toxins, and pigment analyses were collected in acid-washed, sterile bottles, (rinsed three times with *in situ* water before collection) and stored on ice until further processing.

Once transported back to the lab, samples were immediately processed. For dissolved reactive phosphorus (DRP), total dissolved phosphorus (TDP), total dissolved nitrogen (TDN), nitrate, and nitrite, 100 mL of water was filtered through a Whatman glass fiber filter (GF/F) and frozen at −20°C. For TP and total nitrogen (TN), HCl was added to 100 mL of sample to a final concentration of 0.1% and stored at −20°C. Ammonium samples were immediately measured to avoid oxidation during freezing. Chlorophyll-*a* and phycocyanin samples were collected onto GF/F filters and stored in black tubes at −20°C. For community analysis (DNA), samples were filtered onto 0.2 µm polyethersulfone membrane filters (Supor-200; Pall Corporation) and frozen at −20°C until extraction. 20 mL of unfiltered water was preserved in formalin (3% final concentration) and stored at room temperature in the dark for microscopy. An additional 50 mL of unfiltered water was stored at −20°C for toxin analysis.

### Analytical measurements

DRP was measured by the ascorbic acid-molybdenum blue method 4500 P E [38]. Ammonium was measured spectrophotometrically [39]. Nitrate and nitrite were measured individually using high-performance liquid chromatography (HPLC) [40]. TP/TDP and TN/TDN were digested as previously described [41], prior to analysis as for DRP and nitrate. For TDN and TN, the resulting solution was oxidized completely to nitrate and was measured via HPLC as above. Nitrate, nitrite, and ammonium were summed and reported as dissolved inorganic nitrogen (DIN).

Phycocyanin was extracted in 20 mM sodium acetate buffer (pH 5.5) following three freeze-thaw cycles at −20°C and on ice, respectively. The extract was centrifuged and then measured spectrophotometrically at 620 nm with correction at 650 nm [42]. Chlorophyll-*a* (Chl-*a*) was extracted overnight at −20°C in 90% acetone and then measured spectrophotometrically with acid correction [43].

For toxin analysis, whole water samples were lyophilized, resuspended in 5% acetic acid, separated by solid phase extraction (SPE; Bond Elut C18 column, Varian), and eluted in 50% methanol as previously described [44]. Microcystin (MC) variants of leucine (L), arginine (R), and tyrosine (Y) were detected and quantified at the Wisconsin State Lab of Hygiene (SLOH) using electrospray ionization tandem mass spectroscopy (API 3200, MS/MS) after separation by high-performance liquid chromatography (HPLC) [45]. We report only MCLR concentrations since MCYR and MCRR were near the limit of quantification for the sampling period (0.01 µg L^−1^).

### *In situ* N_2_ fixation measurements

N_2_ fixation rates were measured, with some modifications, following the acetylene reduction assay [46]. A fresh batch of acetylene was generated each day before sampling by combing 1 g of calcium carbide (Sigma Aldrich 270296) with 100 mL ddH_2_O. Following sample collection, 1 L of water was concentrated by gentle filtration onto a 47 mm GF/F filter in the field. The filter was then gently washed into a 25 mL serum bottle using the lake water filtrate (final volumes 10 mL aqueous, 15 mL gas). Samples were spiked with 1 mL of acetylene gas and incubated *in situ* for two hours. The assay was terminated with 5% final concentration trichloracetic acid and serum bottles were transported back to the lab. For each sampling period, rates were controlled and corrected for using a series of the following incubated acetylene blanks: 1) 1 mL of acetylene in filtrate alone, 2) 1 mL of acetylene in a killed sample, and 3) 1 mL of acetylene in ddH_2_O. Ethylene formed was measured by a gas chromatograph (GC; Shimadzu GC-8A) equipped with a flame ionization detector (FID), Porapak N column (80/100 mesh, 1/8″OD×6′), and integrator (Hewlett Packard 3396) with N_2_ as the carrier gas (25 mL min^−1^ flow rate). Molar N_2_ fixation rates were estimated using a 1∶4 ratio of N_2_ fixed to ethylene formed [47]. All N_2_ fixation values are reported as integrated photic zone rates of µg N L^−1^ hr^−1^.

### Physical parameters

The light attenuation coefficient (K_d_ [m^−1^]) was calculated using the following equation:
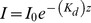
(1)where *I* is the PAR (µmol s^−1^ m^−2^) at depth, *z* (m), and *I*_0_ is the PAR at the lake surface. We calculated daily averages for lake number (L_N_) using high-resolution buoy data and the Lake Analyzer program previously described [48].

### DNA extraction and processing of PC-IGS fragment

DNA was extracted from frozen filters using a xanthogenate-phenol-chloroform protocol previously described [49]. For amplification of the phycocyanin intergenic spacer (PC-IGS) region, we used primers PCαR (5′-CCAGTACCACCAGCAACTAA-3′) and PCβF (5′-GGCTGCTTGTTTACGCGACA-3′, 6-FAM-labelled) and PCR conditions that were previously described [50]. Briefly, each 50 µl reaction mixture contained 5 µl of 10× buffer (Promega, Madison, WI), 2.5 µl of dNTPs (5 mM), 2 µl of forward and reverse primers (10 µM), 2 µl of template DNA, and 0.5 µl of *Taq* DNA polymerase (5 U µl^−1^). Following precipitation with ammonium acetate and isopropanol, the DNA pellet was resuspended in ddH_2_O and digested for 2 hrs at 37°C using the *MspI* restriction enzyme, BSA, and Buffer B (Promega, Madison, WI). The digested product was precipitated and then resuspended in 20 µL of ddH_2_O. 2 µL of final product was combined with 10 µL of formamide and 0.4 µL of a custom carboxy-x-rhodamine (ROX) size standard (BioVentures, Inc).

### Cyanobacterial PC-IGS community fingerprinting and cell counts

We analyzed the cyanobacterial community using an automated phycocyanin intergenic spacer analysis (APISA) similar to the automated ribosomal intergenic spacer analysis (ARISA) previously described [51]. Briefly, this cyanobacterial-specific analysis exploits the variable PC-IGS region of the phycocyanin operon [50]. Following *MspI* digestion, the variable lengths of the PC-IGS fragment can be used to identify subgenus level taxonomic units of the larger cyanobacterial community [49]. The *MspI* fragments were sized using denaturing capillary electrophoresis (ABI 3730×l DNA Analyzer; University of Wisconsin Biotechnology Center (UWBC)). For each sample, triplicate electropherogram profiles were analyzed using GeneMarker® (SoftGenetics) software v 1.5. In addition, a script developed in the R Statistics Environment was used to distinguish potential peaks from baseline noise [52], [53]. Relative abundance data output from this script were created using the relative proportion of fluorescence each peak height contributed per sample. Aligned, overlapping peaks were binned into subgenus taxonomic units [49]. These taxa were named based on the genus and base pair length of the PC-IGS fragment identified (e.g. For Mic215, Mic = *Microcystis* and 215 = 215 base pair fragment). Fragment lengths were matched to an *in silico* digested database of PC-IGS sequences using the Phylogenetic Assignment Tool (https://secure.limnology.wisc.edu/trflp/).

The NTL-LTER program collects biweekly phytoplankton samples between April and September for cell counts and detailed descriptions of the field and laboratory protocols are available online at http://lter.limnology.wisc.edu. When indicated, biomass has been converted to mg L^−1^ using the biovolume calculated during the cell count process and assuming a density equivalent to water.

### Multivariate analyses of the cyanobacterial community

We analyzed the cyanobacterial community based on the PC-IGS fragments using a cluster analysis and canonical correspondence analysis (CCA) to identify the temporal patterns and potential drivers of community change. A similarity matrix was generated from the relative abundance data using the S17 Bray-Curtis coefficient [54] using Primer v6 software [55]. From these data, a hierarchical cluster was created to test community groupings for succession and spatial dispersion. We grouped our samples based on three characteristics—by month, by toxic phase, and by site—to test for differences in the community composition using analysis of similarity (ANOSIM). The “toxic phase” was defined as the time MCLR concentrations were above 1 µg L^−1^ (e.g. day 173 to 224 in 2010).

We used CCA to test for explanatory variables within the cyanobacterial community using CANOCO for Windows software version 4.5.1 [56]. Two CCAs were created to determine whether there was an environmental gradient (e.g. DIN) that explains community differences and if so, whether this environmental gradient was associated with specific operational taxonomic units (OTUs) that are indicative of overall community toxicity. No rare species weighting was performed in either of the CCAs, since all OTUs were present in greater than 10% of our samples and weighting of rare species often has little influence on the significance of CCA [57]. When testing the effects of environmental gradients on specific OTUs, only the top ten OTUs, representing approximately 90% of total fluorescence were included. All environmental variables were log-transformed.

## Results

Lake Mendota experiences strong seasonal trends for temperature, DO, pH, K_d_, and TN, TP, DIN, and DRP concentrations (2010 sites shown in Figure S2) and is dominated by cyanobacteria during the summer months contributing to 80–97% of the biomass between June and September (Figure S3). Overall, phycocyanin increased during the 2010 and 2011 summers, while Chl-*a* slightly decreased. However, pigment analyses were highly variable with a relative standard deviation of up to 25%. A one-way repeated measures analysis of variance (rm-ANOVA) for the three sites sampled in 2010—DH, GA, and UB—showed no significant differences between the major chemical, biological, and physical parameters measured (Table 1). Therefore, for all downstream analyses with the exception of community analysis, all three sites were averaged that year with error bars representing the standard error of the mean, or the propagation of error for N∶P ratios.

**Table 1:** Physical, chemical, and biological parameters measured at 3 Lake Mendota sites.

Parameter	2010						2011	
	Deep Hole (*n* = 14)		Green Acres (*n* = 13)		University Bay (*n* = 14)		Deep Hole (*n* = 23)	
	Mean	Range	Mean	Range	Mean	Range	Mean	Range
Photic zone depth (m)	7.10	4.00–12.0	7.00	5.00–12.0	4.80	4.00–5.00	5.70	3.0–10.5
Attenuation coefficient (m^−1^)	0.74	0.34–1.17	0.79	0.38–0.96	0.77	0.45–0.96	0.70	0.55–0.97
Temperature (°C)	21.5	12.4–26.9	21.9	13.8–26.5	22.7	15.7–27.6	22.7	8.9–27.5
Dissolved oxygen (mg L^−1^)	9.40	7.90–11.8	9.3.0	7.50–11.5	9.10	6.40–11.9	8.60	6.20–12.0
Lake number (dimensionless)	2.47	0.04–7.61	4.02	0.27–9.44	0.27	0.01–1.43	NA	NA
pH (−log [H^+^])	8.30	7.10–9.30	8.3.0	7.40–9.10	8.40	7.60–9.10	NA	NA
Total N (mg L^−1^)	0.78	0.51–1.36	0.79	0.53–1.43	0.72	0.54–1.22	1.24	0.86–1.72
Total dissolved N (mg L^−1^)	0.65	0.36–1.07	0.64	0.38–1.05	0.59	0.36–1.01	NA	NA
Dissolved organic N (mg L^−1^)	0.39	0.29–0.53	0.42	0.33–0.61	0.40	0.27–0.57	NA	NA
Nitrate+nitrite (mg L^−1^)	0.26	0.01–0.63	0.22	0.01–0.65	0.18	BDL-0.67	0.33	0.06–0.91
Total P (mg L^−1^)	0.04	0.02–0.09	0.05	0.02–0.11	0.04	0.02–0.07	0.04	0.02–0.07
Total dissolved P (mg L^−1^)	0.03	0.01–0.07	0.03	0.01–0.08	0.02	0.01–0.07	0.03	0.01–0.06
Dissolved organic P (mg L^−1^)	0.01	0.01–0.02	0.01	0.01–0.02	0.01	BDL-0.03	0.02	BDL-0.04
Soluble reactive P (mg L^−1^)	0.01	BDL-0.06	0.01	BDL-0.06	0.01	BDL-0.06	0.04	BDL-0.05
Chlorophyll-a (µg L^−1^)	6.20	BDL-41.7	3.00	BDL-9.10	7.20	BDL-47.0	32.4	0.80–49.7
Phycocyanin (µg L^−1^)	13.8	1.80–29.5	17.0	3.00–55.3	15.1	2.20–54.2	14.2	3.3–24.9
N_2_ fixation rate (µg N L^−1^ hr^−1^)	0.90	BDL-2.68	0.60	BDL-1.43	0.68	BDL-3.22	0.8	BDL-4.65
MCLR (µg L^−1^)	0.42	0.01–5.24	1.22	BDL-8.43	1.28	BDL-12.8	2.81	BDL-16.1
MCYR (µg L^−1^)	0.02	BDL-0.04	0.01	BDL-0.03	0.01	BDL-0.03	BDL	BDL
MCRR (µg L^−1^)	0.01	BDL-0.06	BDL	BDL	BDL	BDL	BDL	BDL

All values are represented by integrated photic zone samples collected from May 20th–August 31st, 2010. BLD = below the level of detection. NA = not applicable; was not measured in 2011.

In this study, we used the cyanobacteria specific PC-IGS sequence to describe changes in cyanobacterial taxa, and total community, throughout the 2010 and 2011 growing seasons. In total, 100 unique operational taxonomic units (OTUs) were identified in 65 samples (42 in 2010 and 23 in 2011), but only 50 OTUs were above the limit of quantification (LOQ) for relative fluorescence (Figure S4). Eight of these could be assigned to a genus, and at least 5 OTUs were *Aphanizomenon* or *Microcystis* genera. These 5 OTUs accounted for more than half of the total relative fluorescence in the APISA profiles (a proxy for relative abundance). While the majority of PC-IGS OTUs could not be assigned to a genus, probably due to the lack of cultured or sequenced representatives, Aph680 and Mic215 accounted for 21% and 13% of the total APISA profile fluorescence, respectively, in 2010. In addition, there were two OTUs—Aph/Ana/Chr690 and Glo/Chr132—with fragment sequences representative of more than one genus. The Aph/Ana/Chr690 OTU was included when summing total *Aphanizomenon* abundance because *Chroococcus* and *Anabaena* spp. are both rare in the NTL-LTER phytoplankton count records. However, it cannot be determined at this time if that fragment is definitively *Aphanizomenon*. In 2011, Aph680 and Mic215 each represented only 8% of the total relative fluorescence, while Chr352, Mic660, and Aph/Ana/Chr690 represented 12, 12, and 10%, respectively. Microscopic counts of phytoplankton from the NTL-LTER program identified 36 species in 2010 with *Aphanizomenon* and *Microcystis* accounting for 5.8% (53% by biomass) and 3.9% (21% by biomass) of the total cell counts, respectively (Figure S4; Table S1). Twenty-one of the 36 taxa accounted for <1% of the total cell counts. In 2010, there was a good, but insignificant, relationship between total *Aphanizomenon* PC-IGS data and the NTL-LTER cell counts (R = 0.67; *p* = 0.07)(Figure S5). However, there was a significant correlation between the *Microcystis* PC-IGS data and the NTL-LTER cell counts (R = 0.72; *p*<0.05).

**Figure 1:**
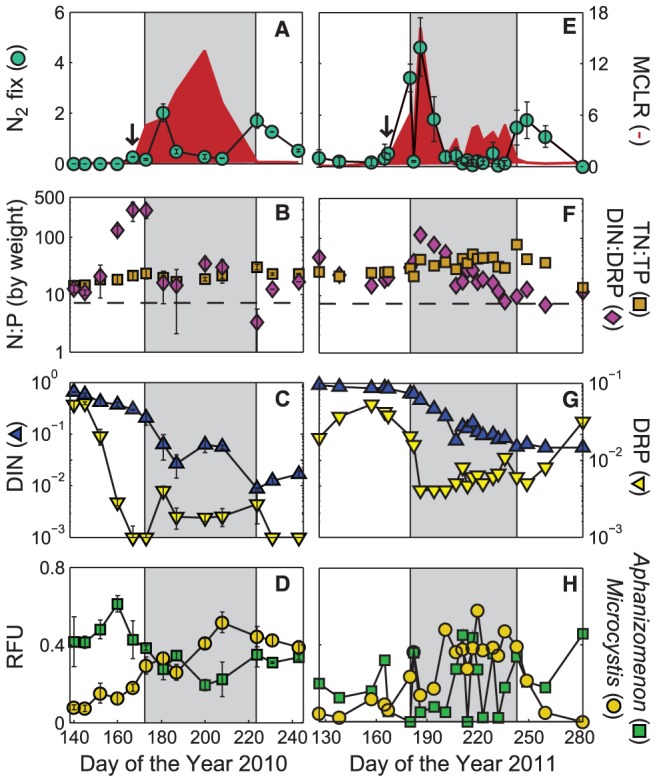
N_2_ fixation rates, microcystin, N and P concentrations, and community dynamics in Lake Mendota from 2010 and 2011. A) 2010 average nitrogen fixation rates (µg N L^−1^ hr^−1^) and microcystin-LR concentrations (µg L^−1^); **B)** 2010 dissolved inorganic nitrogen to dissolved reactive phosphorus ratios (DIN∶DRP) and total nitrogen to total phosphorus (TN∶TP) ratios by weight. The dashed line represents the Redfield reference ratio of 7.2 N∶P by weight [58]; **C)** 2010 DIN and DRP concentrations (mg L^−1^); **D)** 2010 sum of *Aphanizomenon* and *Microcystis* genotypes in relative fluorescence units; **E–H)** Same as A–D, but for 2011, respectively. Note the log scale in panels **B** and **F**. The arrows indicate the first day N_2_ fixation rates were significantly above the limit of detection. For plots **A**, **C**, and **D**, the error bars represent the standard error of the mean between the three sites sampled. For plot **E**, the error bars represent the standard deviation of triplicate samples, and in plot **B**, the error bars represent the propagation of error for N and P between the three sites sampled. The grey box indicates the toxic-phase, which is bound by the pre- and post-toxic phases, where MCLR concentrations were greater than 1 µg L^−1^ (*see* text for details).

Three large N_2_ fixation events (i.e. >1 µg N L^−1^ hr^−1^), presumably driven by *Aphanizomenon*, which represented >31±3% of relative abundance during those events, were observed in 2010 (Figure 1). The first major N_2_ fixation event in early summer (day 181) was the largest with a rate of 2.01±0.35 µg N L^−1^ hr^−1^ with the second and third events (days 224 and 231) measured at 1.70±0.29 and 1.26±0.01 µg N L^−1^ hr^−1^, respectively, occurring in late summer. DIN and DRP concentrations dropped quickly following thermal stratification of the lake and resulted in highly variable DIN∶DRP ratios (by weight) that may suggest short-term N stress and/or limitation (Figure 1). On days where N_2_ fixation rates were high, DIN∶DRP ratios were near the Redfield reference ratio of 7.2 N∶P (by weight) [58]. In the 3 weeks prior to the first major N_2_ fixation event DIN∶DRP ratios were in excess of 100 N∶P. DIN∶DRP ratios increased on day 200 due to a slight increase in nitrate concentrations, but dropped to below 7.2 on the day of the second major N_2_ fixation event.

In 2011, there were six large N_2_ fixation events with three occurring in early summer and three in late summer/fall (Figure 1). Again, the relative abundance of *Aphanizomenon* was >16±10% percent during those events. Most of the error was attributed to *Aphanizomenon* abundance going from 30% to almost 0% during one day in the early summer. The early summer events—days 180, 186, and 194—were much larger in 2011 than in 2010 with rates of 3.45±0.54, 4.65±1.14, and 1.85±0.88 µg N L^−1^ hr^−1^, respectively. Interestingly, DIN∶DRP ratios were quite high (>30∶1 by weight) with DIN concentrations of 0.63, 0.46, and 0.31 mg N L^−1^ during the 3 events (Figure 1). DRP concentrations were also higher in 2011, and neither DIN nor DRP concentrations went to detection in 2011 (Figure 1). Though DIN∶DRP ratios were high in the early summer, they decreased precipitously throughout the summer down to ∼7 (by weight) by day 236. The second set of N_2_ fixation events occurred beginning with day 243, roughly three weeks later than in 2010, and then ceased on day 280 during the onset of fall mixis.

In 2010, MCLR concentrations increased above the LOQ in early summer just days after a minor N_2_ fixation event occurred (day 167, Figure 1). MCLR concentrations increased for the next month, with maximum concentrations occurring in mid-July, and then dropped back down to below the LOQ by day 224 (thus, we defined the toxic phase in 2010 to be between days 173–224). Maximum MCLR concentrations were recorded approximately 20 days after the large early summer N_2_ fixation event, perhaps as *Microcystis* steadily grew over the course of the summer. In total, there were 5 days when observed MCLR concentrations were above the World Health Organization (WHO) level for safe drinking water (1 µg L^−1^) and between the low to moderate risk for recreational swimming, 4 and 20 µg L^−1^, respectively [59]. There were 13 days in 2011 when MCLR concentrations were above the WHO level for safe drinking water. However, only two of those days were significantly above 4 µg L^−1^, both of which occurred just a few days after the first significant N_2_ fixation rates were measured. MCLR concentrations were more variable in 2011, hovering between 1–3 µg L^−1^ for much of the summer, with the toxic phase occurring between days 180 and 243. Average *Aphanizomenon* and *Microcystis* abundances (as measured by APISA) alternated during the sampling period with highest *Microcystis* abundances during times when MCLR concentrations were highest and with *Aphanizomenon* being most abundant during the spring, early summer, and fall when N_2_ fixation was occurring. In 2010, *Aphanizomenon* abundances declined following the first N_2_ fixation bloom, remained low during *Microcystis* dominance, and then increased slightly at the time of the second N_2_ fixation bloom. In 2011, *Aphanizomenon* also declined after the first N_2_ fixation bloom, but it appeared several times throughout the summer as well. In addition, one of the potential *Aphanizomenon* OTUs, AphAnaChr690, greatly increased during the second N_2_ fixation bloom, though it is uncertain if this OTU is represented entirely by *Aphanizomenon*, or perhaps a mix of *Anabaena* and/or *Chroococcus*. On average, for both years, *Aphanizomenon* abundances were highest during the pre- and post-toxic phases and *Microcystis* abundances were highest during the toxic phase.

**Figure 2:**
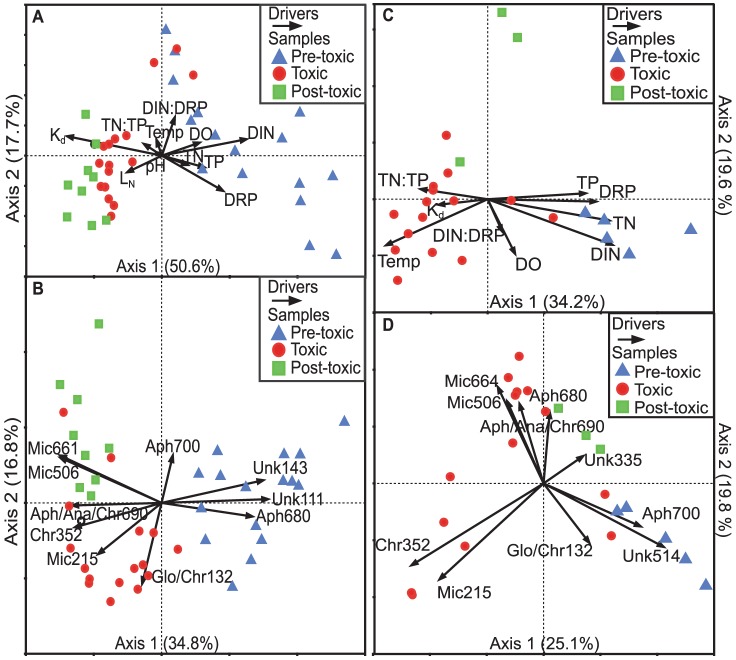
Multivariate plots for 2010 and 2011. A) 2010 canonical correspondence analysis (CCA) of potential environmental drivers on the cyanobacterial community composition assessed using APISA. **B)** 2010 CCA of the cyanobacterial community composition with the top ten most abundant genotypes as drivers of the cyanobacterial community. **C–D)** Same as A and B but for 2011, respectively. Numbers in parentheses represent the amount of variability explained by those axes.

To explore the broader community pattern further, we conducted multivariate statistical analyses with the APISA data, revealing three distinct temporal groupings—pre-toxic, toxic, and post-toxic lake phases. Cluster analysis of the similarity matrices confirmed that all three groupings were significantly different based on analysis of similarity (ANOSIM; *p*<0.001) with R statistics of 0.72, 0.87, and 0.47 for the pre-toxic vs. toxic, pre-toxic vs. post-toxic, and toxic vs. post-toxic groupings, respectively, in 2010 (Figure S6). As with the chemical, physical, and biological variables measured, the three sites sampled—DH, GA, and UB—were not significantly different based on community composition (ANOSIM; *p*>0.8). To assess the relationship between the community and potential environmental drivers, we performed a canonical correspondence analysis (CCA) of the independent variables that may have contributed to the community assemblage (Figure 2, Table S2). DIN and K_d_ had the strongest correlations—r = 0.68 and −0.76, respectively—to the first axis with the DIN gradient pointing toward June samples and K_d_ pointing toward July–August samples. However, interpretation of K_d_ as a driver of community composition is confounded by the fact that cyanobacteria influence K_d_ because they are buoyant and shade the water column at high densities. The DIN∶DRP ratio and DRP were most correlated to the second axis—r = 0.37 and −0.34, respectively; however, the DIN∶DRP gradient correlated with samples transitioning from the pre-toxic to the toxic grouping while DRP correlated with May samples.

Cluster analysis of the 2011 similarity matrices also confirmed that pre-toxic vs. toxic, pre-toxic vs. post-toxic, and toxic vs. post-toxic groupings were significantly different based on analysis of similarity (ANOSIM; *p*<0.05) with R statistics of 0.39, 0.99, and 0.31, respectively (Figure S7). The CCA pattern was very similar in 2011 with DIN concentrations having a strong correlation with the first axis (r = 0.86; Figure 2; Table S4). Temperature also had a strong correlation with the first axis, r = −0.70 as did TN (r = 0.84). Again, the DIN∶DRP ratio corresponded with the transition from the pre-toxic to the toxic phase, r = −0.27 with the second axis, though the relationship was much weaker than in 2010.

We also performed a CCA of the top 10 most abundant OTUs as drivers of the cyanobacterial community (Figure 2, Table S3). Aph680 was highly correlated with the first axis (r = 0.70), which aligned with pre-toxic samples. Mic215 was less correlated with any of the axes (r = −0.49) but aligned with the middle of the toxic phase, while Mic506 and Mic660 were more correlated with the post-toxic phase. Aph700 corresponded with the second axis (r = 0.46), which is indicative of its presence in both pre-toxic and post-toxic phases (Figure 2). In 2011, Aph680 and Aph700 were opposite that of 2010, with Aph700 corresponding to pre-toxic samples and Aph680 corresponding to post-toxic samples. All Mic OTUs corresponded to the toxic phase in 2011 (Figure 2, Table S5). However, Mic215 correlated to the early toxic phase (r = −0.63) while Mic506 and Mic660 correlated to late summer samples—r = −0.22 and −0.27, respectively.

**Figure 3:**
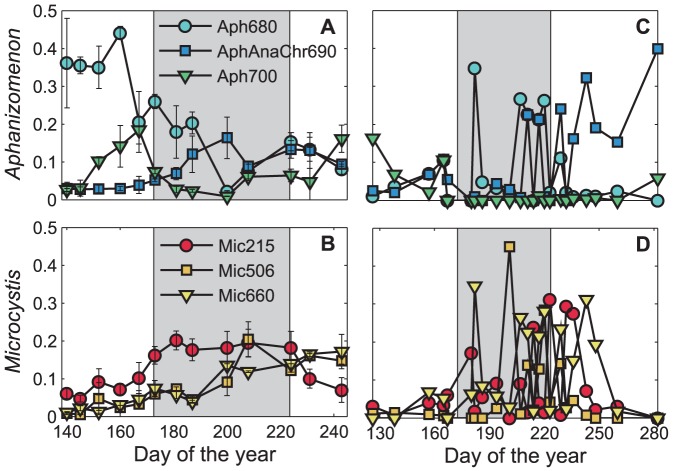
Major genotype time-series from 2010 and 2011 in Lake Mendota. **A)** Two major *Aphanizomenon* genotypes and a third, yet to be distinguished genotype that represents a PC-IGS fragment similar to *Aphanizomenon*, *Anabaena*, and *Chroococcus*. **B)** Three major *Microcystis* genotypes identified. **C–D)** Same as A and B, but for 2011, respectively. In 2010, Mic215 was significantly correlated to MCLR. However, no genotypes were significantly correlated to MCLR in 2011. The grey box indicates the toxic-phase, which is bound by the pre- and post-toxic phases, where MCLR was greater than 1 µg L^−1^ (*see* text for details). The error bars represent the standard error of the mean between genotypes of the three sites sampled.

The abundances of *Aphanizomenon* and *Microcystis* OTUs in APISA profiles were variable throughout the sampling period, with Aph680 and Mic215 making up the majority of the 2010 community during most of the spring and summer (Figure 3). Aph680 comprised approximately 40% of the cyanobacterial community for most of the pre-toxic phase and then declined the rest of the year. However, Aph700 abundance peaked in spring and fall to about 20% of the total community. This trend is also apparent in the Bray-Curtis similarity matrix (Figure S6) and OTU-community CCA (Figure 2). Mic215 increased steadily from pre-toxic to post-toxic phases, approximately 7% to 20%, and then declined as the summer progressed. However, Mic560 and Mic660 increased continuously from approximately 0% to 15% throughout the entire sampling period. Again, this trend is also seen in the OTU-community CCA (Figure 2) as Mic215 aligned to the toxic phase and was also significantly correlated to MCLR concentrations (r = 0.73, *p*<0.005). In 2011, Aph700 was most abundant at the beginning of the survey (∼20% relative fluorescence) but was less than 10% of the community the rest of the year (Figure 3). Aph/Ana/Chr690 made up roughly 40% of the community in the post-toxic phase. Aph680 and all Mic OTUs were highly variable throughout the sampling period. No OTUs correlated with MCLR in 2011, which was also more variable than in 2010.

## Discussion

**Figure 4:**
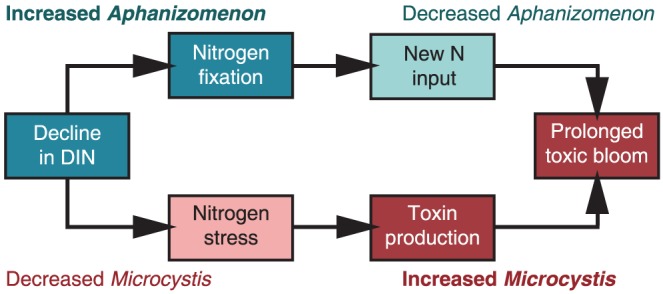
Conceptual diagram of the potential role of nitrogen stress in Lake Mendota, WI. A decline in dissolved inorganic nitrogen (DIN) could have two simultaneous effects: 1) stimulate N_2_ fixation by diazotrophs (e.g. *Aphanizomenon*) that will lead to new N inputs into the photic zone and 2) lead to N starvation in non-N_2_ fixers (e.g. *Microcystis*), which may potentially stimulate toxin production. The new N inputs could then support growth, prolonging toxic blooms of non-N_2_ fixers throughout the growing season.

The current ecological functions of, and environmental triggers/conditions required for, microcystin production in aquatic ecosystems are unknown. We have shown that short-term N stress is important in structuring cyanobacterial communities in a temperate eutrophic lake. We propose that this N stress may also act to stimulate microcystin expression in non-N_2_ fixers such as *Microcystis*, which as been shown to occur in culture-based studies [12]. New N inputs from N_2_ fixation may then prolong toxic cyanobacterial blooms throughout the growing season (Figure 4). In our study, we found that following lake stratification, DIN concentrations dropped precipitously over a period of three weeks in both years, which led to large N_2_ fixation events comprised of mostly *Aphanizomenon*. The influx of new N from *Aphanizomenon* and their subsequent slowed growth led to *Microcystis* blooms coinciding with an increase in MCLR concentrations. In 2010, APISA OTU Mic215 was significantly correlated to this increase. However, in 2011, no OTUs correlated with MCLR. This may be due to the relatively high concentrations of both N and P, allowing for both *Microcystis* and *Aphanizomenon* to coexist at times throughout the summer (Figure 3). In both years, prior to fall mixing, there was a decrease in *Microcystis*, MCLR, and DIN that once again led to N_2_ fixing events. However, the lake quickly transitioned to being dominated by other phytoplankton (e.g. diatoms) once water temperatures dropped and fall mixing occurred. Thus, we hypothesize that N stress plays an important role in eutrophic lakes by structuring the cyanobacterial community each summer.

We suggest that N availability may determine the amount of maximum microcystin production in Lake Mendota. Xu *et al* [60] also suggested that N availability was key to the proliferation of *Microcystis* in Lake Taihu, China. While Lake Taihu is a shallow polymictic lake, Lake Mendota is deep and dimictic. Thus, N inputs from N_2_ fixation are likely the main source of available N to surface waters during the summer. Previous research in Lake Mendota, Wisconsin dating back 90 years support our findings [61], [62], [63], [64], suggesting that this may be an ideal system for studying the impact of variable N and P loading, N_2_ fixation and new N production, community dynamics, and cyanotoxin production in eutrophic lakes. However, we note that this is the first study to report cyanotoxin concentrations in Lake Mendota. Torrey and Lee [62] estimated that N_2_ fixation contributed roughly 7% to the annual TN budget of Lake Mendota. Although this estimate appears small, viewing N_2_ fixation in terms of annual budgets may be misleading, and Torrey and Lee (1976) concluded that this input was large enough to support summer algal growth. Similarly, in our study, we proposed that N_2_-fixation could provide enough new N to sustain a toxic bloom throughout the summer. In addition, Gardner and Lee [63] measured amino acid concentrations in Lake Mendota and found that increases in amino acids were always preceded by *Aphanizomenon* blooms. While the authors attributed this to cell senescence, the release of amino acids during N_2_ fixation has been observed [33], [34].

To corroborate past findings, we estimated the amount of new N produced from the first N_2_ fixation blooms to determine whether they could support *Microcystis* growth and MCLR production. The mean N_2_ fixation rate for June 30^th^ (Day 181) was 2.01±0.35 µg N L^−1^ hr^−1^. Assuming that N_2_ fixation occurs only during the day, we multiplied the hourly rate by 14 hours, which was the period of daylight for that day. We then chose 3 different scenarios, based on cyanobacterial sedimentation and nutrient uptake rates [65], for the amount of new N that could be retained in the epilimnion: 1) 100% of N is retained in the epilimnion due to rapid uptake and low sedimentation rates of *Aphanizomenon*, 2) 50% of new N is incorporated into new cyanobacterial biomass and the rest is exported due to high rates of *Microcystis* sedimentation, and 3) 20% of new N is incorporated into new cyanobacterial biomass due to inefficient uptake and high sedimentation rates. In 2010, the calculated amount of new N produced ranged from 4.6–33 µg N L^−1^ d^−1^. The sum of MCLR produced from Day 181 to Day 208 was 39 µg MCLR L^−1^, which is equivalent to 5.5 µg N L^−1^ based on the ratio of MCLR∶N (997 g MCLR mol^−1^ to 140 g N mol MCLR^−1^). Given conservative estimates, and based on only this one N_2_ fixation event, the amount of new N produced from N_2_ fixation could be sufficient to stimulate and/or support MCLR production throughout the summer. In addition, the maximum *Microcystis* biomass observed during this time was 180 µg L^−1^ on day 200 (NTL-LTER; data not shown). If we assume that N content is roughly 10% of *Microcystis* biomass [66], then our new N estimates would also be sufficient to support some, or all, of the 18 µg N L^−1^ needed for *Microcystis* growth. In 2011, the N_2_ fixation events were much greater than in 2010, as was the amount of MCLR produced. In total, 64 µg MCLR L^−1^, equivalent to 9 µg N L^−1^, was measured over the course of the growing season. Although we do not have *Microcystis* biomass estimates for 2011, the two early summer N_2_ fixation events could have produced between 5.9–56.6 µg N L^−1^ and 7.0–79.8 µg N L^−1^, respectively, which is more than triple that of 2010. However, the large amount of new N added, possibly in excess, may have also allowed *Aphanizomenon* to co-exist to some extent throughout the summer.

**Figure 5:**
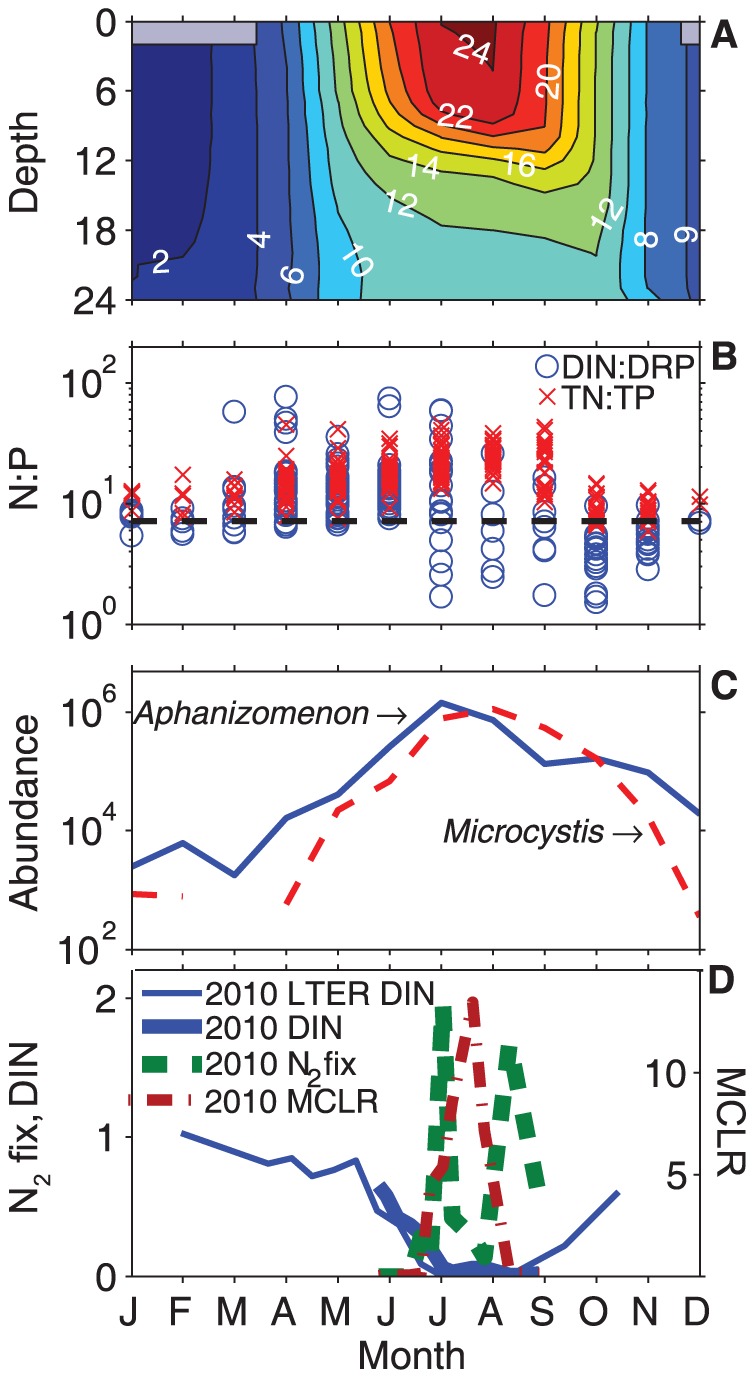
Monthly values from the North Temperate Lakes-Long Term Ecological Research program (1995–2010). **A)** Temperature (°C) profile from 0–24 m depth (averaged across years), with the gray boxes indicating the average ice-on dates, **B)** DIN∶DRP and TN∶TP ratios by weight, for each year. The dashed line represents the Redfield reference ratio of 7.2 N∶P by weight [58], **C)** Total *Aphanizomenon* and *Microcystis* abundances averaged across years (cells mL^−1^), **D)** 2010 DIN from the North Temperature Lakes-Long Term Ecological Research (NTL-LTER) program (mg L^−1^), 2010 DIN from this study (mg L^−1^), and 2010 MCLR concentrations (µg L^−1^) from this study.

Since 1995, the NTL-LTER program has collected phytoplankton cell counts on Lake Mendota, and in those years, *Aphanizomenon* emergence has preceded *Microcystis* in every single year (data not shown). Although this may be due to a number of physiological characteristics (e.g. optimal growth temperature), it does underscore the role N cycling and N_2_ fixation may play in stimulating toxic cyanobacterial blooms, particularly of *Microcystis*. Following ice-off and spring mixing, the phytoplankton community is made up primarily of Bacilliarophyta, Chlorophyta, Chrysophyta, and to a lesser extent, Cyanophyta (Figure S3). However, cyanobacteria clearly dominate (e.g. >90% of phytoplankton biomass) between the months of May and October once surface temperatures increase to >18°C. In particular, *Aphanizomenon* abundance peaks before *Microcystis* by an average of about one month and then *Microcystis* increases until the lake begins to destratify in the fall (Figure 5). Interestingly, high DIN∶DRP ratios correlated more with the N_2_ fixing *Aphanizomenon*, and low DIN∶DRP ratios, which decreased steadily throughout the summer, correlated more with the non-N_2_ fixing *Microcystis*, while the inverse was true for TN∶TP ratios. Dolman et al. [8] also observed that low N∶P ratios did not always correspond to a higher abundance of N_2_-fixing cyanobacteria and warranted that the cyanobacteria should not be treated as a single group. Our results indicate that the cyanotoxin, MCLR, increased during an increase in *Microcystis* abundance when inorganic nitrogen concentrations were very low (as were DIN∶DRP ratios) and TN∶TP ratios were high, but this is only true during the summer. In 2011, DIN and DRP concentrations never approached detection and DIN∶DRP ratios were very high (e.g. >7.2 by weight) in early summer, even when N_2_ fixation occurred. We speculate that this may be due to the rapid decline in ammonium first, triggering N_2_ fixation until enough nitrate is reduced intra-cellularly to shut the process down. Although our ammonium measurements were always near detection, data from the NTL-LTER suggests that this may be true at the onset of lake stratification. The higher nitrate concentrations may have also allowed *Aphanizomenon* to become occasionally abundant throughout the 2011 summer, which wasn't apparent in 2010. In both years, though, a second N_2_ fixing bloom occurred before fall mixing (Figure 1). However, during fall mixing, rapid increases in ammonium and decreasing temperatures are likely to lead the phytoplankton community to shift back to predominantly diatoms (Figure S3). This corresponded with high DIN and DRP concentrations but relatively low DIN∶DRP ratios, since the injection of phosphate from the hypolimnion was relatively greater than that of ammonium plus nitrate. Thus, in the fall, the low DIN∶DRP ratio was a very poor predictor of *Microcystis* abundance.

## Conclusions

We have attempted to link cyanobacterial population dynamics with ecosystem function changes (e.g. N stress and N_2_ fixation) in a temperate, eutrophic lake. More field-scale studies need to be done connecting the functional/physiological dimensions of N metabolism to toxin production in order to assess future water quality scenarios. Thus, it is imperative that our conceptual view of eutrophication be more specific if we are to understand the effects of nutrient loading on more complex changes in phytoplankton population dynamics, bloom development, and toxin production [67], [68]. Our data suggest that N stress plays a major role in structuring cyanobacterial communities in Lake Mendota, Wisconsin—a large, temperate, and dimictic lake. We used an ecosystem approach measuring important structural and functional metrics such as population dynamics, N_2_ fixation rates, and microcystin concentrations allowing us to link intra-annual changes in specific cyanobacterial OTUs. We propose that new N production supports the growth of the potentially toxic cyanobacteria, *Microcystis*, and may be the limiting factor in total toxin production during the growing season. This phenomenon may be important in other freshwater systems that have variable N and P concentrations throughout the growing season.

## Supporting Information

Figure S1 Map of Lake Mendota sites sampled during the 2010 field season (in 2011, only the Deep Hole location was sampled). Maximum depths are displayed in parentheses.
(EPS)

Figure S2 Seasonal trends of major biological, chemical, and physical parameters measured from May 18^th^ to August 31^st^, 2010, in Lake Mendota, WI. Solid line = Deep Hole, dashed line = Green Acres, and dotted line = University Bay locations. None of the parameters were significantly different between sites (*p* values listed).
(EPS)

Figure S3 Major phytoplankton divisions in Lake Mendota, WI, USA. Average monthly biomass (mg L^−1^) for the four major divisions of phytoplankton as measured by the North Temperate Lakes-Long Term Ecological Research program from 1995–2010. Diatoms usually dominate in Spring (>80% abundance) and in fall (>50% abundance), while cyanobacteria clearly dominate during the summer (>90% abundance). Biomass is converted from biovolume measurements made during the cell count process assuming cells are equivalent to the density of water.
(EPS)

Figure S4 Rank abundance curves for DNA fingerprinting and cell counts from Lake Mendota, 2010. A) Over 100 operational taxonomic units (OTUs) were detected by amplifying the cyanobacteria specific phycocyanin intergenic spacer (PC-IGS) region, but only 50 were above the limit of quantification based on relative fluorescence units. The top ten genotypes accounted for 88% of the total abundance in Lake Mendota in 2010. Aph680 and Mic215 were the most abundant and represented 21% and 13% of the total fluorescence, respectively. B) Cell count data from the North Temperate Lakes-Long Term Ecological Research (NTL-LTER) program. Taxa from 36 different species were present in 2010 (Listed in Table S2). The top ten most abundant accounted for 96% of total abundance. *Aphanizomenon flos-aquae* and *Microcystis aeruginosa* represented 5.8% (53% by biomass) and 3.9% (21% by biomass) of the total cells, respectively.
(EPS)

Figure S5 Comparison of Lake Mendota PC-IGS fragments and cyanobacterial cell counts collected from the Deep Hole location in 2010. *Aphanizomenon* PC-IGS fragments (blue-green line) and cell counts (blue-green circles) were well correlated (R = 0.68) but insignificantly (*p* = 0.07). However, *Microcystis* PC-IGS fragments (red line) and cell counts (red squares) were significantly correlated (R = 0.72; *p* = 0.04).
(EPS)

Figure S6 Hierarchical cluster analysis of the Bray-Curtis similarity matrix generated from all samples collected in 2010 (*n* = 42). Analysis of similarity (ANOSIM) between sites showed no significant differences (R = −0.039, −0.043, −0.065 for DH/GA, DH/UB, and GA/UB groups, respectively; *p*>0.8). However, community groupings based on pre-toxic (closed triangle), toxic (open circles), and post-toxic (closed squares) phases were significantly different (R = 0.723, 0.868, and 0.486 for pre-toxic/toxic, pre-/post-toxic, and toxic/post-toxic groupings, respectively; *p*<0.001).
(EPS)

Figure S7 Hierarchical cluster analysis of the Bray-Curtis similarity matrix generated from all samples collected in 2011 (*n* = 23). Community groupings based on pre-toxic (closed triangle), toxic (open circles), and post-toxic (closed squares) phases were significantly different (R = 0.386 (*p* = 0.006), 0.990 (*p* = 0.018) and 0.305 (*p* = 0.04) for pre-toxic/toxic, pre-/post-toxic, and toxic/post-toxic groupings, respectively).
(EPS)

Table S1 List of all 36 cyanobacterial taxa identified in 2010 by cell counts. All data are from the North Temperate Lakes-Long Term Ecological Research (NTL-LTER) program and are available online at http://lter.limnology.wisc.edu.
(EPS)

Table S2 2010 results of canonical correspondence analysis (CCA) with environmental variables as drivers of the cyanobacterial community. All values are significant (*p*<0.001) based on a Monte-Carlo simulation test of 1000 permutations.
(EPS)

Table S3 2010 results of canonical correspondence analysis (CCA) with cyanobacterial genotypes as drivers of the cyanobacterial community. All values are significant (*p*<0.001) based on a Monte-Carlo simulation test of 1000 permutations.
(EPS)

Table S4 2011 results of canonical correspondence analysis (CCA) with environmental variables as drivers of the cyanobacterial community. All values are significant (*p*<0.001) based on a Monte-Carlo simulation test of 1000 permutations.
(EPS)

Table S5 2011 results of canonical correspondence analysis (CCA) with cyanobacterial genotypes as drivers of the cyanobacterial community. All values are significant (*p*<0.001) based on a Monte-Carlo simulation test of 1000 permutations.
(EPS)

## References

[1] Carpenter SR, Bolgrien D, Lathrop RC, Stow CA, Reed T, et al (1998) Ecological and economic analysis of lake eutrophication by nonpoint pollution. Australian Journal of Ecology 23: 68–79.

[2] Dodds WK, Bouska WW, Eitzmann JL, Pilger TJ, Pitts KL, et al (2009) Eutrophication of US freshwaters: analysis of potential economic damages. Environmental Science & Technology 43: 12–19. 19209578 10.1021/es801217q

[3] Palmer MA, Febria CM (2012) The heartbeat of ecosystems. Science 336: 1393–1394. 22700910 10.1126/science.1223250

[4] Scott JT, McCarthy MJ (2010) Nitrogen fixation may not balance the nitrogen pool in lakes over timescales relevant to eutrophication management. Limnology and Oceanography 55: 1265–1270.

[5] Schindler DW, Hecky RE, Findlay DL, Stainton MP, Parker BR, et al (2008) Eutrophication of lakes cannot be controlled by reducing nitrogen input: Results of a 37-year whole-ecosystem experiment. Proceedings of the National Academy of Sciences of the United States of America 105: 11254–11258. 18667696 10.1073/pnas.0805108105 PMC2491484

[6] Carpenter SR, Caraco NF, Correll DL, Howarth RW, Sharpley AN, et al (1998) Nonpoint pollution of surface waters with phosphorus and nitrogen. Ecological Applications 8: 559–568.

[7] Paerl HW (1988) Nuisance phytoplankton blooms in coastal, estuarine, and inland waters. Limnology and Oceanography 33: 823–847.

[8] Dolman AM, Rücker J, Pick FR, Fastner J, Rohrlack T, et al (2012) Cyanobacteria and cyanotoxins: the influence of nitrogen versus phosphorus. PLoS ONE 7: e38757. 22719937 10.1371/journal.pone.0038757 PMC3376147

[9] Oh H-M, Lee SJ, Jang M-H, Yoon B-D (2000) Microcystin production by Microcystis aeruginosa in a phosphorus-limited chemostat. 66: 176–179. 10.1128/aem.66.1.176-179.2000 PMC91802 10618220

[10] Watanabe MF, Oishi S (1985) Effects of environmental factors on toxicity of a cyanobacterium (Microcystis aeruginosa) under culture conditions. 49: 1342–1344. 10.1128/aem.49.5.1342-1344.1985 PMC238555 3923932

[11] Pattanaik B, Wulff A, Roleda MY, Garde K, Mohlin M (2010) Production of the cyanotoxin nodularin-A multifactorial approach. Harmful Algae 10: 30–38.

[12] Ginn HP, Pearson LA, Neilan BA (2010) NtcA from Microcystis aeruginosa PCC 7806 is autoregulatory and binds to the microcystin promoter. Applied and Environmental Microbiology 76: 4362–4368. 20453121 10.1128/AEM.01862-09 PMC2897459

[13] Vezie C, Rapala J, Vaitomaa J, Seitsonen J, Sivonen K (2002) Effect of nitrogen and phosphorus on growth of toxic and nontoxic Microcystis strains and on intracellular microcystin concentrations. Microbial Ecology 43: 443–454. 11953809 10.1007/s00248-001-0041-9

[14] Utkilen H, Gjølme N (1995) Iron-stimulated toxin production in Microcystis aeruginosa. 61: 797–800. 10.1128/aem.61.2.797-800.1995 PMC167340 7574617

[15] Sevilla E, Martin-Luna B, Vela L, Bes MT, Fillat MF, et al (2008) Iron availability affects mcyD expression and microcystin-LR synthesis in Microcystis aeruginosa PCC7806. Environmental Microbiology 10: 2476–2483. 18647335 10.1111/j.1462-2920.2008.01663.x

[16] Jähnichen S, Long BM, Petzoldt T (2011) Microcystin production by Microcystis aeruginosa: Direct regulation by multiple environmental factors. Harmful Algae 12: 95–104.

[17] Sivonen K (1990) Effects of light, temperature, nitrate, orthophosphate, and bacteria on growth of and hepatotoxin production by Oscillatoria-agardhii strains. Applied and Environmental Microbiology 56: 2658–2666. 2125814 10.1128/aem.56.9.2658-2666.1990 PMC184825

[18] Orr PT, Jones GJ (1998) Relationship between microcystin production and cell division rates in nitrogen-limited Microcystis aeruginosa cultures. Limnology and Oceanography 43: 1604–1614.

[19] Long BM, Jones GJ, Orr PT (2001) Cellular microcystin content in N-limited Microcystis aeruginosa can be predicted from growth rate. Applied and Environmental Microbiology 67: 278–283. 11133456 10.1128/AEM.67.1.278-283.2001 PMC92564

[20] Sivonen K, Jones GJ (1999) Cyanobacterial toxins. In: Chorus I, Bartram J, editors. Toxic cyanobacteria in water: a guide to their public health consequences. London: Spon. pp. 41–111.

[21] Downing TG, Meyer C, Gehringer MM, van de Venter M (2005) Microcystin content of Microcystis aeruginosa is modulated by nitrogen uptake rate relative to specific growth rate or carbon fixation rate. Environmental Toxicology 20: 257–262. 15892070 10.1002/tox.20106

[22] Alexova R, Haynes PA, Ferrari BC, Neilan BA (2011) Comparative protein expression in different strains of the bloom-forming cyanobacterium *Microcystis aeruginosa* . Molecular & Cellular Proteomics 10. 10.1074/mcp.M110.003749 PMC3186190 21610102

[23] Oh H-M, Lee SJ, Kim J-H, Kim H-S, Yoon B-D (2001) Seasonal variation and indirect monitoring of microcystin concentrations in Daechung Reservoir, Korea. 67: 1484–1489. 10.1128/AEM.67.4.1484-1489.2001 PMC92758 11282594

[24] Wicks RJ, Thiel PG (1990) Environmental factors affecting the production of peptide toxins in floating scums of the cyanobacterium Microcystis aeruginosa in a hypertrophic African reservoir. Environmental Science & Technology 24: 1413–1418.

[25] Kotak BG, Lam AKY, Prepas EE, Kenefick SL, Hrudey SE (1995) Variability of the hepatoxin microcystin-LR in hypereutrophic drinking water lakes1. Journal of Phycology 31: 248–263.

[26] Kotak BG, Zurawell RW, Prepas EE, Holmes CF (1996) Microcystin-LR concentration in aquatic food web compartments from lakes of varying trophic status. Canadian Journal of Fisheries and Aquatic Sciences 53: 1974–1985.

[27] Rinta-Kanto JM, Konopko EA, DeBruyn JM, Bourbonniere RA, Boyer GL, et al (2009) Lake Erie Microcystis: Relationship between microcystin production, dynamics of genotypes and environmental parameters in a large lake. Harmful Algae 8: 665–673.

[28] Vezie C, Brient L, Sivonen K, Bertru G, Lefeuvre JC, et al (1998) Variation of microcystin content of cyanobacterial blooms and isolated strains in Lake Grand-Lieu (France). Microbial Ecology 35: 126–135. 9541549 10.1007/s002489900067

[29] Wilhelm SW, Farnsley SE, LeCleir GR, Layton AC, Satchwell MF, et al (2011) The relationships between nutrients, cyanobacterial toxins and the microbial community in Taihu (Lake Tai), China. Harmful Algae 10: 207–215.

[30] Li JH, Laurent S, Konde V, Bédu S, Zhang CC (2003) An increase in the level of 2-oxoglutarate promotes heterocyst development in the cyanobacterium Anabaena sp. strain PCC 7120. Microbiology 149: 3257–3263. 14600238 10.1099/mic.0.26462-0

[31] Xu H, Paerl HW, Qin B, Zhu G, Gao G (2010) Nitrogen and phosphorus inputs control phytoplankton growth in eutrophic Lake Taihu, China. Limnology and Oceanography 55: 420–432.

[32] Dugdale RC, Goering JJ (1967) Uptake of new and regenerated forms of nitrogen in primary productivity. Limnology and Oceanography 12: 196–206.

[33] Glibert PM, Bronk DA (1994) Release of dissolved organic nitrogen by marine diazotrophic cyanobacterium, Trichodesmium spp. Applied and Environmental Microbiology 60: 3996–4000. 16349432 10.1128/aem.60.11.3996-4000.1994 PMC201927

[34] Capone DG, Ferrier MD, Carpenter EJ (1994) Amino acid cycling in colonies of the planktonic marine cyanobacterium Trichodesmium thiebautii. Applied and Environmental Microbiology 60: 3989–3995. 16349431 10.1128/aem.60.11.3989-3995.1994 PMC201926

[35] Agawin NSR, Rabouille S, Veldhuis MJW, Servatius L, Hol S, et al (2007) Competition and facilitation between unicellular nitrogen-fixing cyanobacteria and non-nitrogen-fixing phytoplankton species. Limnology and Oceanography 52: 2233–2248.

[36] Gondwe MJ, Guildford SJ, Hecky RE (2008) Planktonic nitrogen fixation in Lake Malawi/Nyasa. Hydrobiologia 596: 251–267.

[37] Lenes JM, Heil CA (2010) A historical analysis of the potential nutrient supply from the N_2_ fixing marine cyanobacterium Trichodesmium spp. to Karenia brevis blooms in the eastern Gulf of Mexico. Journal of Plankton Research 32: 1421–1431.

[38] Greenberg AE, Clesceri LS, Eaton AD (1992) Standard methods for the examination of water and wastewater. Washington, EUA: American Public Health Association.

[39] Solórzano L (1969) Determination of ammonia in natural waters by the phenol hypochlorite method. Limnology and Oceanography 14: 799–801.

[40] Flowers JJ, He S, Yilmaz S, Noguera DR, McMahon KD (2009) Denitrification capabilities of two biological phosphorus removal sludges dominated by different ‘Candidatus Accumulibacter’ clades. Environmental Microbiology Reports 1: 583–588. 20808723 10.1111/j.1758-2229.2009.00090.x PMC2929836

[41] White AE, Karl DM, Björkman K, Beversdorf LJ, Letelier RM (2010) Production of organic matter by *Trichodesmium* IMS101 as a function of phosphorus source. Limnology and Oceanography 55: 1755–1767.

[42] Demarsac NT, Houmard J (1988) Complementary chromatic adaptation - physiological conditions and action spectra. Methods in Enzymology 167: 318–328.

[43] Tett P, Kelly MG, Hornberger GM (1975) Method for spectrophotmetric measurement of chlorophyll-a and pheophytin-a in benthic microalgae. Limnology and Oceanography 20: 887–896.

[44] Harada K-I, Matsuura K, Suzuki M, Oka H, Watanabe MF, et al (1988) Analysis and purification of toxic peptides from cyanobacteria by reversed-phase high-performance liquid chromatography. Journal of Chromatography A 448: 275–283. 10.1016/s0021-9673(01)84589-1 3147286

[45] Eaglesham GK, Norris RL, Shaw GR, Smith MJ, Chiswell RK, et al (1999) Use of HPLC-MS/MS to monitor cylindrospermopsin, a blue-green algal toxin, for public health purposes. Environmental Toxicology 14: 151–154.

[46] Stewart WDP, Fitzgerald GP, Burris RH (1967) In situ studies on N2 fixation using the acetylene reduction technique. Proceedings of the National Academy of Sciences of the United States of America 58: 2071–2078. 5237501 10.1073/pnas.58.5.2071 PMC223907

[47] Jensen BB, Cox RP (1983) Direct measurements of steady-state kinetics of cyanobacterial N_2_ uptake by membrane-leak mass-spectrometry and comparisons between nitrogen-fixation and acetylene-reduction. Applied and Environmental Microbiology 45: 1331–1337. 16346272 10.1128/aem.45.4.1331-1337.1983 PMC242459

[48] Read JS, Hamilton DP, Jones ID, Muraoka K, Winslow LA, et al (2011) Derivation of lake mixing and stratification indices from high-resolution lake buoy data. Environ Model Softw 26: 1325–1336.

[49] Miller TR, McMahon KD (2011) Genetic diversity of cyanobacteria in four eutrophic lakes. FEMS Microbiology Ecology 78: 336–348. 21707672 10.1111/j.1574-6941.2011.01162.x

[50] Neilan BA, Jacobs D, Goodman AE (1995) Genetic diversity and phylogeny of toxic cyanobacteria determined by DNA polymorphisms within the phycocyanin locus. Applied and Environmental Microbiology 61: 3875–3883. 8526499 10.1128/aem.61.11.3875-3883.1995 PMC167692

[51] Yannarell AC, Kent AD, Lauster GH, Kratz TK, Triplett EW (2003) Temporal patterns in bacterial communities in three temperate lakes of different trophic status. Microbial Ecology 46: 391–405. 12904915 10.1007/s00248-003-1008-9

[52] Jones SE, Cadkin TA, Newton RJ, McMahon KD (2012) Spatial and temporal scales of aquatic bacterial beta diversity. Frontiers in Microbiology 3. 10.3389/fmicb.2012.00318 PMC3431545 22969757

[53] Jones SE, McMahon KD (2009) Species-sorting may explain an apparent minimal effect of immigration on freshwater bacterial community dynamics. Environmental Microbiology 11: 905–913. 19040451 10.1111/j.1462-2920.2008.01814.x

[54] Legendre P, Legendre L (1998) Numerical ecology, 2nd Edition (Developments in Environmental Modelling, Vol. 20). Amsterdam: Elsevier. 853 p.

[55] Clarke KR, Gorley RN (2006) PRIMER V6: User Manual/Tutorial. Plymouth, UK: PRIMER-E.

[56] Ter Braak CJF, Šmilauer P (2002) CANOCO Reference Manual and CanoDraw for Windows User's Guide: Software for Canonical Community Ordination (version 4.5). Ithaca, NY: Microcomputer Power.

[57] Legendre P, Gallagher E (2001) Ecologically meaningful transformations for ordination of species data. Oecologia 129: 271–280. 28547606 10.1007/s004420100716

[58] Redfield AC (1934) On the proportions of organic derivations in sea water and their relation to the composition of plankton; Daniel RJ, editor: University Press of Liverpool. 16 p.

[59] WHO (1996) Guidelines for drinking-water quality. Volume 2: Health criteria and other supporting information. 2nd Edition. Geneva Switzerland: World Health Organization. xvi + 973 p.

[60] Xu Y, Wang GX, Yang WB, Li RH (2010) Dynamics of the water bloom-forming Microcystis and its relationship with physicochemical factors in Lake Xuanwu (China). Environmental Science and Pollution Research 17: 1581–1590. 20512630 10.1007/s11356-010-0345-8

[61] Birge EA, Juday C (1922) The inland lakes of Wisconsin. The plankton. I. Its quantity and chemical composition. Wisconsin Geological Survey Bulletin. pp. 222.

[62] Torrey MS, Lee GF (1976) Nitrogen fixation in Lake Mendota, Madison, Wisconsin. Limnology and Oceanography 21: 365–378.

[63] Gardner WS, Lee GF (1975) The role of amino acids in the nitrogen cycle of Lake Mendota. Limnology and Oceanography 20: 379–388.

[64] Fallon RD, Brock TD (1980) Plankton blue-green algae: production, sedimentation, and decomposition in Lake Mendota, Wisconsin. Limnology and Oceanography 25: 72–86.

[65] Brock TD (1985) A eutrophic lake - Lake Mendota, Wisconsin. New York: Springer-Verlag. 308 p.

[66] Gerloff GC, Skoog F (1957) Nitrogen as a limiting factor for the growth of Microcystis aeruginosa in southern Wisconsin lakes. Ecology 38: 556–561.

[67] Cloern J (2001) REVIEW: Our evolving conceptual model of the coastal eutrophication problem. Marine Ecology Progress Series 210: 223–253.

[68] Glibert PM, Burkholder JM (2011) Harmful algal blooms and eutrophication: “strategies” for nutrient uptake and growth outside the Redfield comfort zone. Chinese Journal of Oceanology and Limnology 29: 724–738.

